# Tumefactive Crohn's Disease Mimicking Colon Carcinoma in a Young Female: A Case Report

**DOI:** 10.7759/cureus.52221

**Published:** 2024-01-13

**Authors:** Ali Z Ansari, Sania Razzak, Srihita Patibandla, Sarthak Kumar, Sahar Hafeez, Kurt Kratz

**Affiliations:** 1 Pathology, William Carey University College of Osteopathic Medicine, Hattiesburg, USA; 2 Clinical Pathology, William Carey University College of Osteopathic Medicine, Hattiesburg, USA; 3 Pathology, Merit Health Wesley, Hattiesburg, USA

**Keywords:** anatomical pathology, ulcerative colitis (uc), colon carcinoma, inflammatory bowel disease, crohn's disease (cd)

## Abstract

Tumefactive Crohn’s disease is a rare form of Crohn’s disease that may mimic colon carcinoma macroscopically. This case report describes a 28-year-old female who presented with right-sided abdominal pain and a palpable abdominal mass that had persisted for over a month. Multiple hospitalizations failed to provide an accurate diagnosis until an exploratory laparotomy revealed that the "mass" was the cecum and a perforated ascending colon. A partial right colectomy was performed, sending the specimen for biopsy. The microscopic description showed dense and confluent chronic inflammation in the colonic mucosa and wall, extending to the serosa in some regions. The infiltration comprised lymphocytes and plasma cells, with an admixture of some neutrophils. Aphthous mucosal ulcerations, intramural fissures, and fistulas were present. Immunostains for pan-keratin demonstrated no intramural epithelial elements. The characteristics of this lesion represent tumefactive Crohn’s disease. This case highlights the key microscopic characteristics that pathologists look for when differentiating Crohn's disease from colon carcinoma in a patient presenting with abdominal pain and a colon mass.

## Introduction

Inflammatory bowel disease (IBD) is an umbrella term describing a variety of chronic gastrointestinal disorders, mainly involving two subtypes: Crohn’s disease and ulcerative colitis. While both conditions share common inflammatory origins, there are significant differences in terms of clinical presentations and pathological characteristics. For both conditions, patients often present with colicky abdominal pain, fecal urgency, and fecal incontinence [1]. Crohn’s disease is characterized by transmural inflammation affecting any segment of the gastrointestinal tract, most commonly the ileum and proximal colon [2]. In contrast, ulcerative colitis is characterized by inflammation that is confined to the mucosal layer of the colon. Crohn's disease on pathology is characterized by granulomas and discontinuous lesions, whereas ulcerative colitis is characterized by continuous lesions [3]. These differences in pathology are reflected in clinical presentations, with Crohn’s disease often causing transmural complications such as strictures and fistulas, and ulcerative colitis most commonly causing localized mucosal ulceration and bloody diarrhea [4]. Periods of flare and remission during the course of the disease are a common feature in both subtypes of IBD. Symptoms are shown to begin gradually and then progress over the course of several weeks [5]. Consequently, accurate differentiation between these two conditions is crucial for guiding appropriate treatment strategies and screenings and improving patient symptoms.

Crohn's disease is most commonly seen in the Western developed world mostly in North America, northern Europe, and New Zealand [6]. Its incidence has a bimodal distribution with the onset occurring most frequently between ages 15 to 30 years and 40 to 60 years old [6]. It is more prominent in urban than rural areas [6,7]. Among the various presentations of Crohn’s disease, tumefactive Crohn’s disease is a rare occurrence. This atypical subtype presents as localized masses within the gastrointestinal tract, often mimicking the appearance of colorectal carcinoma. The exact frequency or prevalence of tumefactive Crohn’s disease is not well-documented in the medical literature due to its rarity. In general, typical Crohn’s disease is more common and affects a larger number of individuals, with estimates of prevalence varying by region.

Crohn’s disease and colon cancer are both medical conditions that can be difficult to differentiate clinically. Both disorders can share many similar features. A goal in the surveillance of IBD is to detect the possibility of dysplasia. They both can have significant colon involvement, be heavily associated with inflammation, and have slight treatment overlap depending on stage and severity [3]. In this report, a case of tumefactive Crohn’s disease will be described detailing the clinical course of a young female patient who presented with persistent abdominal pain and rebound tenderness.

## Case presentation

A 28-year-old female with a past medical history of cesarean section with tubal ligation and colitis diagnosed about a month ago presented to the emergency department with the complaint of right-sided abdominal pain which has gotten worse over two weeks. Over the past two years, the patient reported to the hospital several times with complaints of abdominal pain, nausea, and vomiting and had been treated with a course of levofloxacin and metronidazole. During her last visit to the hospital a month ago, she finally had been diagnosed with colitis through a computed tomography (CT) scan and was put on another course of metronidazole and levofloxacin. Initially, she noticed an improvement in her symptoms but after a couple of weeks, the abdominal pain returned with a higher intensity which brought her back to the hospital. The patient also admits to being able to feel a "mass" in her right lower quadrant at times. She had a planned colonoscopy scheduled after her last hospital visit but was unable to tolerate her current pain levels, so she decided to return to the emergency department before she could make it to her appointment. She denied any associated symptoms such as fever, chills, and shortness of breath. She also denied noticing any blood in her stool or passing dark stools. There was no history of inflammatory or neoplastic gastrointestinal disorders in her family. The patient claimed to be a non-smoker and non-alcoholic.

**Figure 1:**
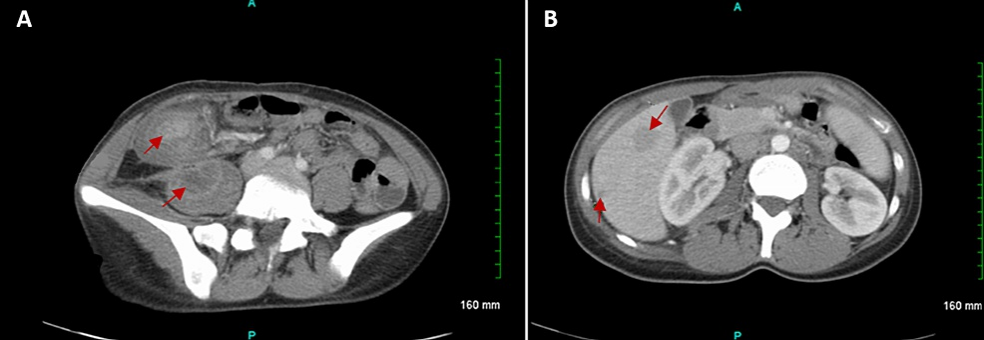
CT scan of the abdomen and pelvis with contrast (A) Abnormal circumferential enhancement edematous changes involving portion of the ascending colon and the cecum with direct involvement of the terminal ileum and the appendix (red arrow). Trace free fluid in the pelvis. Heterogenous enhancement involving the psoas muscle measuring 4.2 cm x 2.8 cm suggestive of abscess (red arrow). (B) Incidental findings of two hypoenhancing liver lesions (red arrows), to be followed up with elective outpatient liver MRI after the acute process is over.

A CT scan of the abdomen showed abnormal edematous lesions involving portions of the ascending colon as well as the cecum with direct involvement of the terminal ileum and appendix, and trace-free fluid in the pelvis (Figure 1A). The process extended to heterogenous enhancement involving the psoas muscle on the right, where a lesion measuring 4.2 cm x 2.8 cm is present (Figure 1A). There were also incidental findings of two hypo-enhancing lesions, with the largest lesion being adjacent to the gallbladder measuring 2.7 cm × 1.9 cm, and a smaller lesion located lateral to the right lobe of the liver (Figure 1B). No abnormalities were detected in the spleen, pancreas, gallbladder, kidney, or adrenal glands. No acute bony lesions were identified. Physical examination revealed a small external hemorrhoid and no rectal masses. Lab work completed in the emergency department showed a mild electrolytes imbalance, and mild anemia, with no evidence of urinary infection. The hemoccult stool test was negative. Procalcitonin was less than 0.05, and CRP was elevated at 9.70. CEA and CA 19-9 tumor markers were within normal ranges at 0.8 and 24 respectively. The unremarkable lab tests, along with the patient's age and overall appearance of the CT scan findings were more in support of an inflammatory rather than a neoplastic process, likely appendicitis or a retrocecal abscess. This was further supported as the patient did endorse pain and symptoms appeared to be resolving when given the antibiotics piperacillin and tazobactam (Zosyn). A colonoscopy was not indicated at the time due to the patient having rebound tenderness in the right lower quadrant requiring immediate operative management. After consulting with the general surgery department to confirm the diagnosis, a decision of exploratory laparotomy with possible colectomy was made to attempt to drain what appears to be an abscess. The patient was shifted to the surgery ward and after receiving a dose of piperacillin and tazobactam (Zosyn) was placed on Nil Per Oral (NPO) status. 

On day two of admission, the patient underwent an exploratory laparotomy. Upon inspection of the abdomen, it was discovered that what initially was thought to be a mass in the right lower quadrant was in fact the cecum and ascending colon. Following the identification of a small cecal perforation on the posterior surface, right partial colectomy and anastomosis were performed with surgical staples. With the anastomosis complete, the psoas abscess was then opened and the loculations were broken. Cultures were obtained and tested positive for gram-positive cocci. The resected specimen composed of cecum and appendix along with a small portion of terminal ileum was sent to the pathology department. The patient was shifted to the ward and was given appropriate post-operative care. The following day, the patient was started on a soft diet which she was able to tolerate. She was later advanced to solid foods without any complications. She was able to pass flatus and the abdominal pain was under control. Once the patient became clinically stable, she was discharged to complete a course of levofloxacin and follow-up in the gastroenterology outpatient clinic.

A pathology report from surgery revealed an attached portion of the terminal ileum measuring 4.5 cm in length and 1 cm in diameter, as well as an attached appendix measuring 5.7 cm in length and 0.6 cm in diameter. There were abundant adhesions located on the serosal surface of the cecum. Upon opening the specimen, the bowel wall in the cecum was noted to be abnormally thick, with some regions reaching up to 1 cm. The mucosa had a roughened appearance that was both irregular and hyperemic. On examination, the ileocecal valve appeared constricted and measured less than 0.5 cm in diameter, with the distal portion exhibiting irregular rugate folds. The terminal ileum exhibited areas of furrowing and irregular mucosa. Due to the abnormal appearance of the cecal mucosa, it was difficult to discern whether the lesions were due to a neoplastic process or just from chronic inflammation.

**Figure 2:**
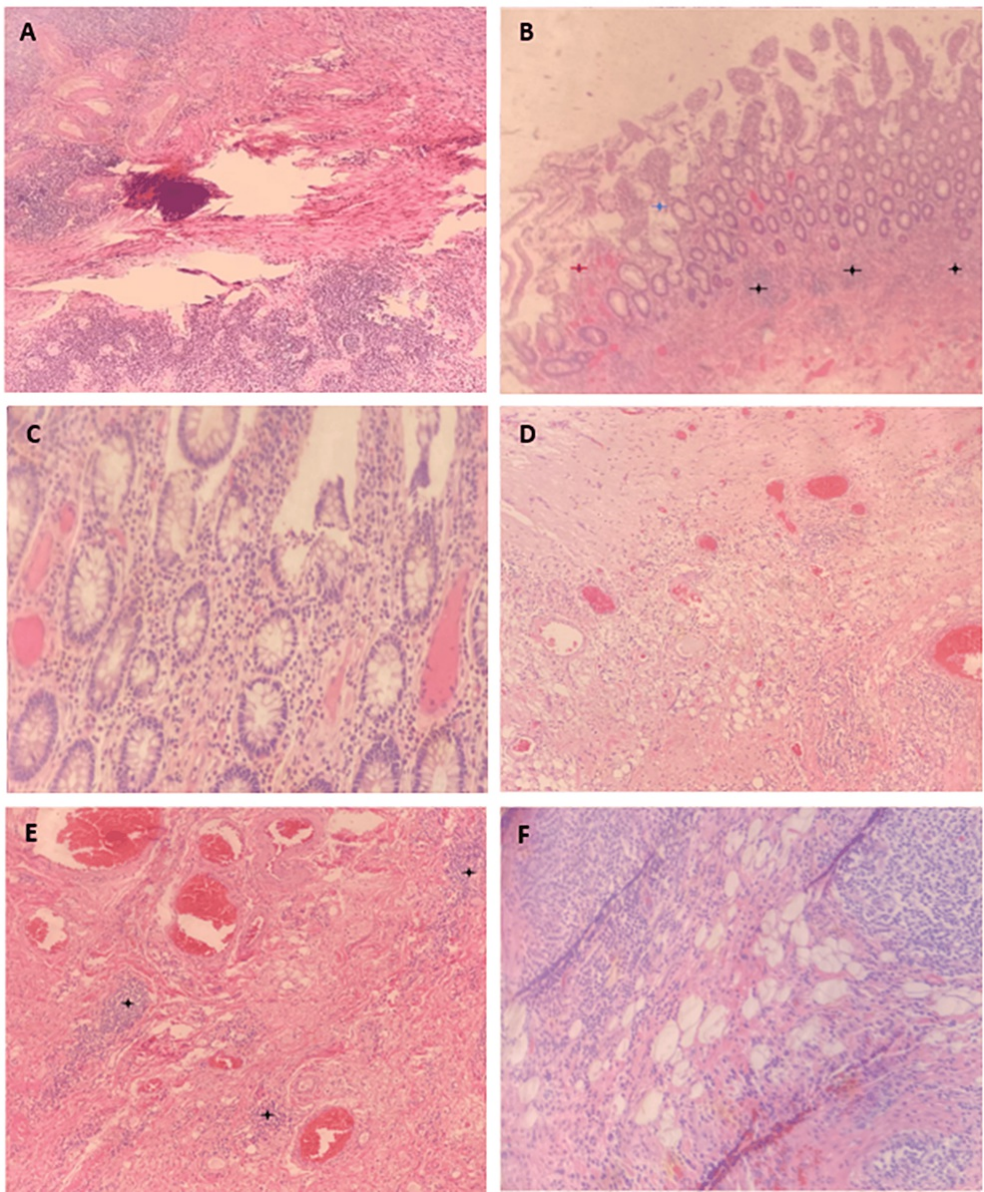
Histological photomicrographs of right colectomy biopsy specimens (A) Cecal wall photomicrograph showing intramural fissure and transmural inflammation. (B) Cecal mucosa close to the ileocecal valve showing aphthous ulceration (red star), crypt destruction (blue star), and crypt abscesses (black stars). (C) Photomicrograph of lamina propria showing a large number of plasma cells with an admixture of neutrophils and eosinophils indicating disease flare. (D) Photomicrograph of hyperemic serosa with a large number of inflammatory cells. (E) Muscularis externa showing dilated blood vessels and small epithelioid granulomas (black stars). (F) Pericolonic adipose tissue showing fat necrosis and lymphoid tissue hyperplasia.

Sections of the specimen were taken and submitted for analysis by two different pathologists. The sections submitted included surgical margins, blue-inked serosa underlying abnormally thickened bowel wall, irregular mucosa and serosa that demonstrated adhesions, and an irregular small bowel as it relates to the ileocecal valve and cecum. Enlarged lymph nodes were also noted in the peri-colonic adipose tissue. A pan-keratin stain showed no infiltrating carcinoma or intramural epithelial elements. However, there was extensive abscess formation, areas of ulceration, and possible evidence of a sinus (Figures 2A, 2B). The appendiceal wall demonstrated marked thickening, serositis, adhesions, fat necrosis, and focal marked chronic inflammation. There were areas of dense and confluent chronic inflammation located in the colonic mucosa and wall that extended to the serosa in various regions (Figures 2A-2F). The infiltrate was composed of lymphocytes and plasma cells, with an admixture of neutrophils (Figures 2C, 2D, 2F). Additional findings included aphthous mucosal ulcerations, intramural fissures, and fistulas (Figures 2A, 2B). No evidence of atypical epithelia was found. It was concluded that the lesion represented tumefactive Crohn’s disease, which has been shown to present in a localized fashion that can imitate the radiographic and gross features of a gastrointestinal carcinoma.

## Discussion

IBDs are chronic and recurrent conditions affecting various parts of the gastrointestinal tract, encompassing two main disorders: Crohn’s disease and ulcerative colitis [1,8]. These disorders are characterized by multifactorial pathogenesis, involving genetic predisposition, defects in the gut epithelial barrier, dysregulated immune responses, and environmental factors, collectively shaping disease presentation [8]. Crohn’s disease and ulcerative colitis diverge when it comes to their clinical presentations, epidemiology, and disease progression, leading to distinct management strategies [5,8]. Despite these differences, the initial stages of both conditions often present overlapping features on tissue biopsies, necessitating meticulous interpretation guided by clinical presentation for an accurate diagnosis and appropriate treatment plan [5,8]. 

Ulcerative colitis is predominantly confined to the large bowel, with the inflammatory process limited to the mucosa. The mucosa has a friable, granular appearance which may progress to denudation or deep penetration, with superficial ulcers that can progress to involving the muscularis mucosae in severe, untreated cases [3]. Crohn’s disease, on the other hand, can affect any part of the gastrointestinal tract. About one-third of patients have small bowel involvement, especially the terminal ileum, another 20% have only colon involvement, and about 50% have involvement of both the colon and small bowel [3,6]. In Crohn’s disease, the inflammatory process extends through the entire thickness of the intestinal wall with evidence of serosal and peri-intestinal fat involvement [6]. Crohn's disease has been reported to be the cause of 0.2% to 1.8% of cases of appendicitis requiring appendectomies [7]. These surgeries typically include removal of the appendix, cecum, and terminal ileum [7]. Affected bowel segments may display hyperemic serosa with inflammatory exudate, accompanied by the development of serosal adhesions over time [8,9]. The resected specimen of the cecum, appendix, and terminal ileum in our patient showed multiple adhesions on the serosal surface with increased thickness of the cecal wall. Microscopically, the colonic lesions showed signs of dense and confluent inflammation extending through the entire thickness of the cecal wall and the inflammatory infiltrate mostly comprised of lymphocytes and plasma cells. The presence of intramural fissures further confirmed the diagnosis of Crohn’s as it is known that with multiple relapses, Crohn’s can progress from a moderate inflammatory condition to a severe penetrating disease with stricture and fistula formation [6,10]. In some areas, the evidence of mucosal sloughing and active cryptitis with neutrophilic infiltrate indicated towards disease flare state.

Small bowel adenocarcinoma (SBA) is a rare malignancy that originates from the epithelial lining of the small intestine. Risk factors for SBA include hereditary mutations, celiac disease, and IBD [11,12]. Recent insights suggest distinctions between Crohn’s disease-induced SBA and de-novo SBA, involving mutations of different genes and impacting a younger demographic [12]. Crohn’s disease-induced SBA most commonly involves the terminal ileum. Crohn’s disease-induced SBA typically presents with intermittent abdominal pain, weight loss, and nausea. Delays in diagnosis result in intestinal obstruction in 33% of cases [13]. The presence of features like fibrosis and stricture formation that can be present in both Crohn’s disease as well as in SBA makes the diagnosis quite challenging. Longstanding Crohn’s disease causes inflammation and scar tissue formation, resulting in the mutation of tumor suppression genes and chromatin remodeling that increase the risk of carcinogenesis [12].

Patients with IBD carry an increased risk of colorectal cancer (CRC) [9,14]. Studies have shown that a high proportion of patients with colorectal cancer had an antecedent history of poorly characterized bowel symptoms, such as frequent loose bowel movements and vague abdominal pain, or a diagnosis of irritable bowel syndrome [8]. According to one study, the risk of CRC is approximately three times greater in people with IBD than in people with no IBD and early-onset CRC (18-49 years) is more common in males than in females [15]. It is observed that IBD patients diagnosed with cancer often show evidence of multiple mucinous tumors which appear as solid lesions close to the areas of chronic inflammation [16]. Many clinicopathological features of colorectal carcinoma complicating Crohn’s disease or ulcerative colitis are directly comparable and suggest a potential common underlying mechanism. Recent clinical studies have indicated that the increased cancer risk seen in inflammatory bowel disease is probably due to the underlying chronic mucosal inflammation, a high epithelial cell turnover, and an increased rate of sporadic mutations [17].

Even though the gender and demographics of the patient were not supportive of CRC or SBA, a relatively short history of IBD with isolated right colonic lesions on CT scan and the distortion of ileal mucosa forced us to rule out the possibility of any malignancy. Histopathologic examination of the ileocolic junction showed evidence of villous destruction and an increased number of goblet cells, but no degree of dysplasia which is the most reliable marker of increased risk of malignancy, or proliferation of atypical epithelial cells was seen. As an admixture of neutrophils in the depths of the crypts was noted along with the lymphocytes and plasma cells, it was suggested that the patient was in the flare stage of Crohn’s disease. The regenerating epithelium in active IBD often shows changes overlapping with those observed in true dysplasia, therefore, the neovascularization with no evidence of nuclear atypia or reversal of nucleus-to-cytoplasmic ratio, and the loss of delineation between the layers of the intestinal wall with multiple epithelioid granulomas in this case is thought to be due to tumefactive Crohn’s disease [9]. When looking at the pathophysiology behind tumefactive Crohn’s disease, the major characterizing features are cyclical periods of flares and remissions in which inflammation is a key factor. It has been shown that there are alterations in the inflammatory process that are caused by changes in the innate defense of intestinal mucosa through the expression of adhesion molecules and metalloproteins. Due to this, there is an increased Th1 response leading to the inflammatory cytokines IL-12 and TNF-alpha [17].

Surgical bowel resection is indicated in patients with Crohn’s disease who have bowel perforation, abscess, hemorrhage, fistula, neoplasm, or severe inflammation that is refractory to medical management [18]. In this case, surgery was indicated due to the presence of cecal perforation. After the exploratory laparotomy, a significant improvement in the symptoms of the patient was noted. Standard post-operative care was given and upon discharge, the patient was able to tolerate and advance her diet without any complications. However, as Crohn's disease can affect any part of the gastrointestinal tract, surgical resection is not curative. Further treatment, for example with biologics, is required for ongoing management.

## Conclusions

This case highlights the importance of considering tumefactive Crohn's disease in the differential diagnosis of young patients presenting with abdominal pain and what appears to be a colonic mass. Evaluation of patients with persistent abdominal pain should be emphasized to identify potential underlying causes, such as Crohn’s disease or ulcerative colitis. In this case, the patient underwent an exploratory laparotomy for appendicitis and abscess, with a right partial colectomy due to cecal perforation, leading to significant symptom improvement. Through histological analysis of the specimen obtained from the patient, it could be confirmed that this was a case of tumefactive Crohn’s disease, rather than the presence of a colonic neoplasm. Understanding the microscopic differences between these two conditions is important for pathologists in making an accurate diagnosis that would guide further post-operative management.
